# Interspecific Competition and Intraspecific Facilitation Shape Coastal Dune Shrub Responses to Experimental Drought

**DOI:** 10.3390/plants14172663

**Published:** 2025-08-26

**Authors:** María Zunzunegui, Mari Paz Esquivias, Mari Cruz Díaz Barradas, Juan B. Gallego-Fernández, Leonor Álvarez-Cansino

**Affiliations:** Departamento de Biología Vegetal y Ecología, Facultad de Biología, Apartado 1095, 41080 Sevilla, Spain; esquiviasmp@gmail.com (M.P.E.); diaz@us.es (M.C.D.B.); galfer@us.es (J.B.G.-F.); leonor@us.es (L.Á.-C.)

**Keywords:** biomass allocation, plant–plant interaction, *Retama monosperma* (L.) Boiss, shoot water potential, *Thymus carnosus* Boiss, leaf stable isotopes

## Abstract

We investigated how water restriction affects plant–plant interactions in two Mediterranean shrubs, *Thymus carnosus* Boiss and *Retama monosperma* (L.) Boiss, to test whether intra- and interspecific interactions between these species respond differently to drought. A greenhouse experiment was conducted with 5-month-old seedlings under three interaction types (interspecific, isolation, and intraspecific). After a 7-month growth phase, a water restriction treatment was imposed, and shoot water potential and photochemical efficiency of chlorophyll were monitored weekly. Biomass allocation and stable isotope composition were analysed at the end of the experiment. *Retama* plants growing alone exhibited the highest relative elongation rate (9.6 cm day^−1^ per plant), whereas for the combinations involving *Thymus*, the highest relative elongation rate occurred under intraspecific competition (3.63 cm day^−1^ per plant). Results showed a negative effect of *Retama* on *Thymus* regarding drought response, while *Thymus* exhibited an intraspecific facilitation effect, improving growth and reducing water stress. Although *Thymus* produced less biomass than *Retama*—with *Retama* producing over 2 g root biomass per plant compared to 0.25 g in *Thymus* and >7 g aboveground biomass versus 2.7 g in *Thymus*—it maintained better physiological response to drought than *Retama*, where all combinations involving *Retama* showed water potential below −2.3 MPa in both species. These findings reveal contrasting strategies: *Retama* prioritises rapid growth but is drought-sensitive, whereas *Thymus* benefits from intraspecific facilitation that enhances drought tolerance. Our results highlight how drought can alter the balance between competition and facilitation in plant interactions, with implications for Mediterranean plant communities’ dynamics under climate change.

## 1. Introduction

Intraspecific and interspecific interactions control the composition of plant communities and can affect community attributes, such as species diversity or resistance to invasion [1,2]. However, multiple factors can alter these plant–plant interactions, including climate conditions, nutrient availability [3], and soil microbial communities. Among these drivers of environmental change, water availability plays a crucial role. In this sense, extreme drought events—expected to become more frequent and intense in the Mediterranean region [4,5]—can significantly influence belowground processes, ultimately modifying plant–plant interactions and reshaping community dynamics [1,6,7].

Mediterranean ecosystems, like other water-limited environments, experience pulses of water availability and discrete precipitation with drought periods, making them especially sensitive to the increased frequency of these extreme drought events [8,9,10]. Rainfall variability and unpredictability impose strong constraints on plants, significantly affecting individual survival [11] and community dynamics [12]. In Mediterranean dune ecosystems, vegetation is highly dependent on rainfall patterns and water availability, with a seasonality strongly marked by hot, dry summers [13]. Climate predictions point to a generalised decrease in precipitation for the Mediterranean region by 2050, with fewer rainy days concentrated more in winter and longer dry periods between rainfall events [4,5,14,15], as has already been observed in recent years. According to meteorological records, arid climates (type B according to Köppen) in the Iberian Peninsula have doubled their extent, increasing from 10% to 21% of the surface area, mainly at the expense of temperate climates (Type C climates), which have decreased [16]. Therefore, Mediterranean ecosystems are highly vulnerable to climate-induced changes in water availability [15,17], which may alter species distribution and plant–plant interactions [18].

Plant–plant interactions play a key role in the establishment of new species, particularly in the seedling stage, which is one of the most critical phases influencing species persistence within plant communities [19,20]. Seedlings and juveniles are generally more sensitive to dehydration than seeds or adult plants [9,21]. Consequently, many species establish under the canopy of other plants, where higher humidity levels promote survival, a phenomenon known as the “nurse effect” [22]. In addition, differences in seedlings’ size and ontogenetic stage can affect the outcome of interspecific competition [23].

The net balance of species interactions is determined by the combination of positive and negative influences, both direct and indirect [24,25]. Studying each factor in isolation is crucial for understanding its specific effects and impact at different levels, such as the root system, canopy, water availability, or shade. This approach can offer valuable insights into the role of individual interactions and their consequences on plant performance and community dynamics.

The functional balance theory [26,27] states that the belowground fraction’s growth is promoted in response to water or mineral nutrient scarcity, while the aerial parts of the plant are favoured under conditions of low light or CO_2_ [28,29]. This aligns with the biomass allocation theory proposed by [30]. Belowground resource competition can be intense and has been associated with spatial segregation of roots [31,32], as plants alter their root production in the presence of other plants [33,34,35]. Roots can either avoid other roots [36,37] or, conversely, proliferate in their presence [38], interfering with or inhibiting the growth of neighbouring roots [39,40,41], using secondary compounds [41]. Therefore, knowing how species function belowground is crucial in determining their competitive ability against other plants. Additionally, fine-root interference plays a critical role in determining a species’ competitive success [42,43].

We selected two native woody species representative of Mediterranean shrub vegetation that naturally co-exist: *Thymus carnosus* Boiss and *Retama monosperma* (L.) Boiss. *T. carnosus* is a protected species with medicinal and culinary uses, while *R. monosperma* is described as a key species in ecosystem restoration and erosion control, as it has been used for dune stabilisation [44]. This species also possesses medicinal properties [16,45]. *R. monosperma* can be considered an expansive species, as it is spreading and increasing in abundance within its original range [46]. Several studies have reported negative impacts of expansive species on plant communities, which can be comparable to those of non-native species [47]. In fact, the expansion of *R. monosperma* has posed significant conservation challenges for several native *T. carnosus* populations [48,49], prompting efforts to reduce *Retama* density to improve *T. carnosus* habitats [50]. Studying these two species and their interactions is crucial to understanding how plants respond to drought and competition and also sheds light on the potential threat that *R. monosperma* poses to the endangered *T. carnosus*, with important implications for their physiological responses, competitive dynamics, and conservation.

Under natural conditions, the interaction of adult individuals of *T. carnosus* with *R. monosperma* has been observed to have a negative net effect on *Thymus* [48,51,52]. However, it is still unknown whether this is the result of the allocation of biomass towards the photosynthetic part to the detriment of the root fraction due to the effect of the shade of the *R. monosperma* canopy, according to the theory of [30], or whether there is a direct competition between the roots of both species for underground resources. Despite the documented negative interaction, the influence of drought on this relationship and belowground competition between the species has not been thoroughly explored. These previous studies [48,51,52] provide a solid basis for exploring how drought influences species dynamics and whether belowground competition for resources plays a role in their responses to water stress and survival under climate change scenarios. Given the projected escalation in both the frequency and severity of drought events [53], assessing their effect on plant–plant interactions within the context of global climate change is crucial. By conducting controlled drought experiments, we can evaluate how water stress affects plants during a critical developmental phase—the active growth period—while minimising the influence of other environmental stressors, such as high temperatures or nutrient scarcity. This approach enhances our understanding of how shifts in rainfall patterns may affect ecosystems under future climate scenarios.

Within this aim, a greenhouse experiment was set up to evaluate the impact of drought on plant–plant interactions. We aimed to assess whether the ability of the focal species to resist and recover after drought was differently affected by the intra- or interspecific interactions (competition or facilitation) between the species. Specifically, our objectives were to determine (1) the effect of intra- or interspecific interaction on biomass allocation patterns in *Thymus carnosus* and *Retama monosperma* under two levels of water availability and (2) the effect of intra- or interspecific interaction on the physiological performance of plants under two levels of water availability. The experiment was conducted with five-month-old seedlings of both species (to eliminate the shading effect of *Retama* on *Thymus*) in three interaction scenarios: interspecific, intraspecific, and isolated. After seven months of co-growth, plants were subjected to a five-week water restriction period, avoiding exposure to high temperatures to simulate a water deficit scenario outside the summer season. This approach is particularly relevant, as previously mentioned, given that climate change projections indicate an increased frequency of droughts during the growing season (winter), which could have significant ecological consequences. Plant responses to competition were analysed through growth, physiological, and biomass allocation measurements.

Given the already known competition for water between the two species and the negative effect on *T. carnosus* under natural conditions [51], the starting hypothesis is that the presence of *R. monosperma* will negatively affect the water status and physiological performance of *T. carnosus* under greenhouse conditions. Following the functional balance theory, the growth of the belowground fraction would be promoted at the expense of the aerial parts under drought conditions. Studying how these species respond to drought and interact with each other can have practical implications for ecosystem management and conservation.

## 2. Results

### 2.1. Growth Pattern Before the Water Restriction Experiment

**Figure 1:**
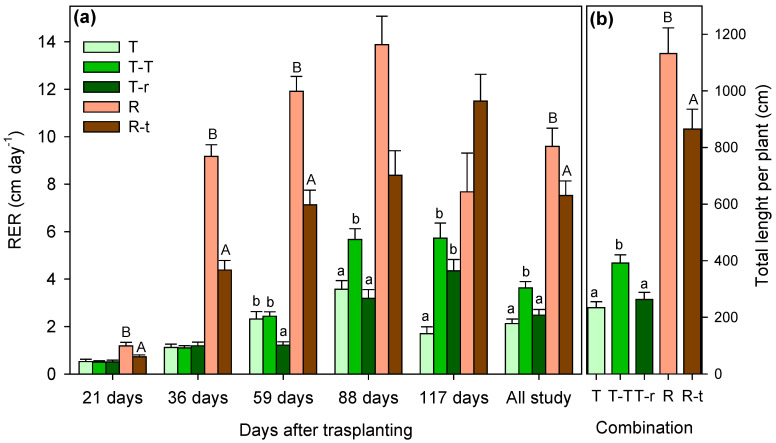
Relative elongation rate (RER) between successive measurement dates (**a**) and total elongation (**b**) considering the sum of the elongation of all the branches of a plant over the entire study period (mean + SE). Significant differences between species combinations are indicated by letters above the columns (lowercase for *Thymus carnosus* Boiss and uppercase for *Retama monosperma* (L.) Boiss; one-way ANOVA, Tukey’s test for *Thymus*; *p* < 0.05). No letters indicate the absence of significant differences.

**Figure 2:**
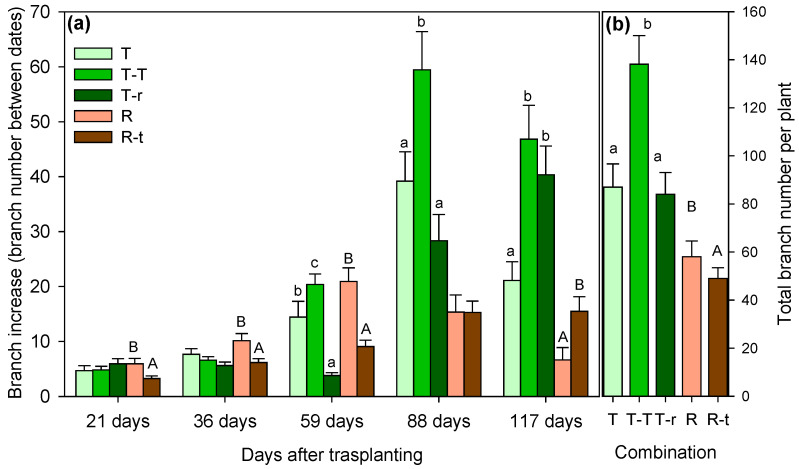
Increase in the number of branches (ΔR) between successive measurement dates (**a**) and total number of branches (**b**) at the end of the study period (mean + SE). Significant differences between species combinations are indicated by letters above the columns (lowercase for *Thymus* Boiss and uppercase for *Retama monosperma* (L.) Boiss; Kruskal–Wallis followed by Mann–Whitney U test, *p* < 0.05).

Under well-watered conditions, growth patterns differed significantly among species and competition treatments. In *Thymus*, relative elongation rate (RER) measured 117 days after transplanting was significantly higher in the T–T combination (3.63 cm day^−1^) compared to T (2.31 cm day^−1^) and T-r (2.48 cm day^−1^) (Figure 1a). Branching followed a similar pattern, with T–T plants consistently producing more branches (138 branches) (Figure 2). Significant effects of time and combination × time interactions were also detected (Table 1).

In *Retama*, isolated plants (R) initially showed greater RER (9.59 cm day^−1^) and branching than R–t plants (growing with *Thymus*). However, by day 117, RER differences had disappeared, and R–t individuals exhibited higher branching.

Total elongation values (Figure 1b) represent the sum of all branch lengths per plant over the 117-day growth period. In R plants, total elongation averaged 1132 ± 544 cm per plant, based on a mean of 59 branches, each averaging 19.3 cm in length. In contrast, T–T plants showed a total elongation of 392 ± 171 cm per plant with 138 branches averaging 3.1 cm per plant (Figure 1b). Overall, the greatest length and branching occurred in T–T (for combinations involving *Thymus*) and R (for combinations involving *Retama*) plants (Figure 1b and Figure 2b).

When species were compared, *Retama* showed a significantly higher relative elongation rate (RER) than *Thymus*, reaching a mean of nearly 14 cm day^−1^ per plant. In contrast, *Thymus* never exceeded 6 cm day^−1^ per plant, even in the T–T combination (Figure 1). The opposite pattern was observed for branching. *Thymus* consistently produced more branches, particularly in the T–T treatment, with a maximum average of 138 branches per plant. Even in the T–r combination, *Thymus* reached a minimum of 86 branches, while *Retama* averaged 59 branches per plant.

**Figure 3:**
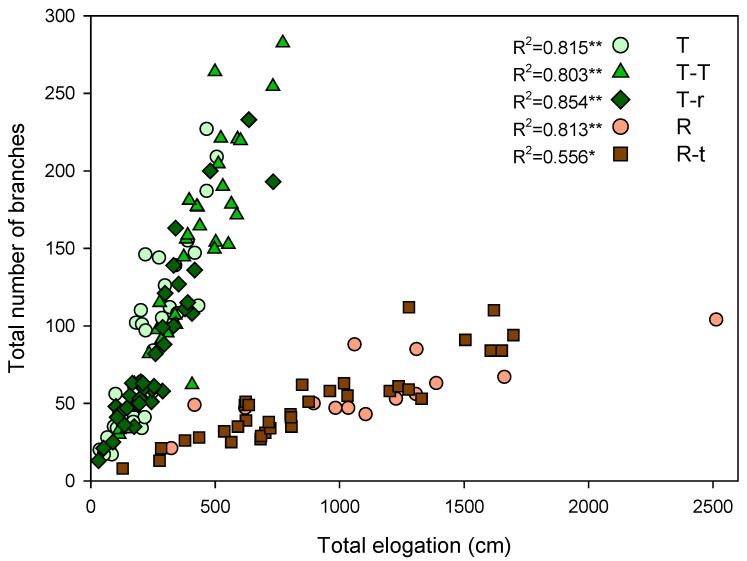
Correlation between the sum of the elongation of all plant branches and the number of branches at the end of the study period. Spearman’s r is next to the legend (* *p* < 0.01 and ** *p* < 0.001). Each point represents an individual.

A strong positive correlation between total elongation and branch number was found in both species, indicating that individuals with more branches also achieved greater total shoot growth (Figure 3). This pattern was consistent across interaction treatments, although the slopes differed between species. These results suggest species-specific growth strategies that remained stable under both intra- and interspecific competition.

### 2.2. Water Restriction Experiment

**Figure A1:**
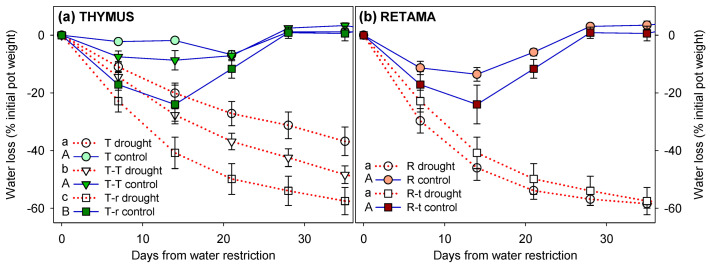
Evolution of water loss during the water restriction treatment, expressed as the percentage decrease in pot weight relative to the initial weight on day 0 (pre-treatment) and relative to field capacity (FC), reflecting cumulative loss through evapotranspiration during drought treatment (day 0 = pre-treatment) (mean ± SE). Significant differences between combinations over the entire treatment period are indicated next to the legend with different letters (lowercase for drought pots, uppercase for control pots; ANOVA, *p* < 0.05).

After the onset of water restriction, drought pots lost moisture rapidly. Final weight reduction was 38% in T, 51% in T-T, and 58% in both R and T-r pots (which are the same pots as R-t, labeled according to the species measured) (Figure A1). Significant differences in weight loss were observed between drought and control pots across all combinations after the 6-week treatment period. In control pots, no significant differences in weight loss were found among combinations. Under drought conditions, however, T-r and R pots exhibited significantly greater weight loss than T or T-T pots. Additionally, T-r control pots showed a sharper weight loss during the first two weeks compared to other combinations, until rewatering to FC was applied.

#### 2.2.1. Shoot Water Potential

**Figure 4:**
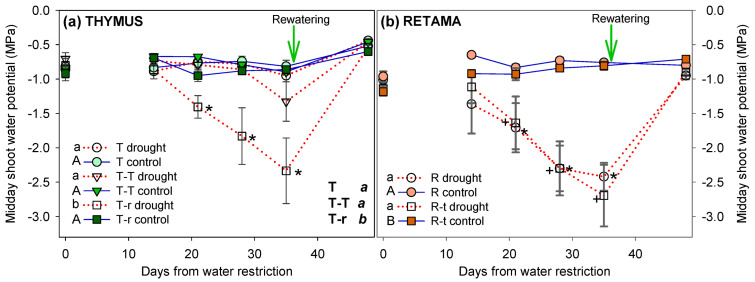
Evolution of midday shoot water potential (mean + SE) in *Thymus carnosus* Boiss (**a**) and *Retama monosperma* (L.) Boiss (**b**) combinations throughout the water restriction treatment (Day 0 = pre-drought; Day 49 = recovery). Significant differences between combinations over the entire treatment period are indicated next to the legend with different letters (lowercase for drought individuals and uppercase for control individuals, analysed using two-way ANOVA; overall significance analysed by three-way ANOVA is indicated in uppercase black letters; *p* < 0.05). Asterisks denote significant pairwise differences between control and water restriction treatments for each measurement time point. The arrow marks the recovery irrigation event.

During the drought period, no significant differences in Ψ_m_ were observed among control combinations. In drought pots, all combinations involving *Retama* showed a significantly progressive decline in Ψ_m_, reaching values below –2 MPa in both *Thymus* and *Retama*. In contrast, *Thymus* individuals growing alone (T) or under intraspecific competition (T–T) did not show significant differences between control and drought treatments by the end of the experiment (Figure 4). After rewatering, Ψm increased across all combinations, with no significant differences remaining between treatments. The three-way ANOVA for *Thymus* revealed a significant effect for the three factors: competition, time, and watering (Table 2). For *Retama*, competition had no significant effect, suggesting a similar response to water restriction whether growing alone or in competition with *Thymus* (Figure 4b).

#### 2.2.2. Photochemical Efficiency

**Figure 5:**
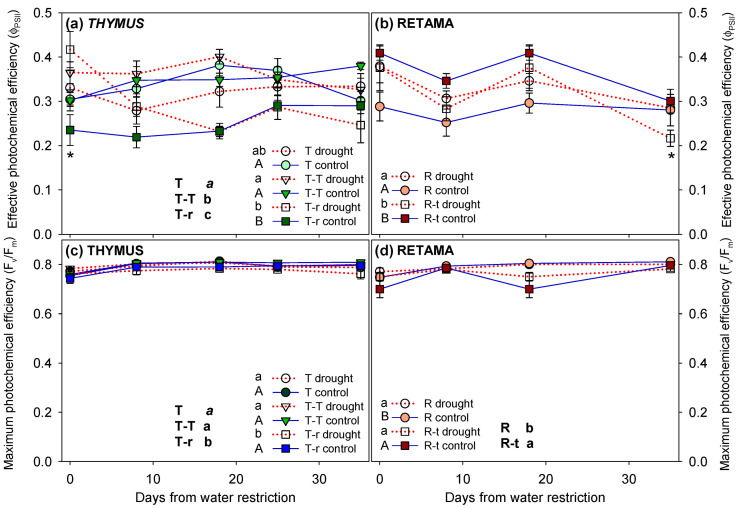
Effective photochemical efficiency (Φ_PSII_) and maximum photochemical efficiency (Fv/Fm) of *Thymus carnosus* Boiss, T, T-T, and T-r (**a**,**c**), and *Retama monosperma* (L.) Boiss combinations (mean + SE), R and R-t (**b**,**d**), throughout the drought treatment (day 0 = before water restriction). Significant differences between combinations over the entire treatment period are indicated next to the legend with different letters (lowercase for drought individuals and uppercase for control individuals, analysed using two-way ANOVA; overall significance analysed by three-way ANOVA is indicated in uppercase black letters; *p* < 0.05). Asterisks denote significant pairwise differences between control and water restriction treatments for each measurement time point.

Significant differences were found in photochemical efficiency (Φ_PSII_) and maximum photochemical efficiency (F_v_/F_m_) in *Thymus* across competition treatments, as well as in all interactions between competition, watering, and time (Figure 5a,c, Table 2). The results indicate that the drought response of *Thymus* plants depended on the type of competition they were subjected to (Figure 5a). Plants in the T-T combination showed the highest Φ_PSII_ (0.380) and F_v_/F_m_ (0.789) values, whereas T-r plants exhibited the lowest (0.246 and 0.762, respectively). In *Retama*, Φ_PSII_ and F_v_/F_m_ also varied across competition treatments (Figure 5b,d, significant differences indicated next to the legend with different letters). However, no significant differences in F_v_/F_m_ were detected between drought and control treatment in *Retama* across the different competition scenarios. Notably, F_v_/F_m_ values never dropped below a mean of 0.74 in any treatment. In summary, the fluorescence measurements revealed that the type of plant–plant interaction (particularly interspecific competition between *Thymus* and *Retama*) had a stronger effect on photochemical parameters than the water restriction treatment itself.

**Figure A2:**
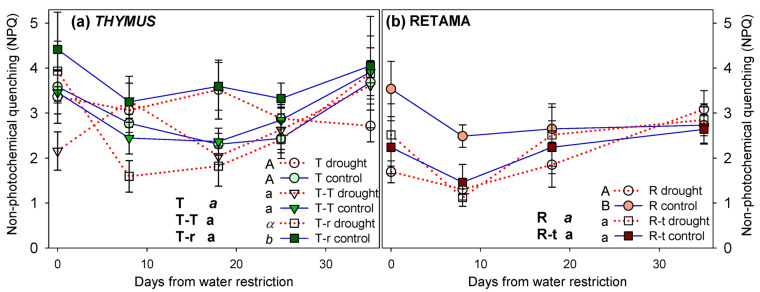
Non-photochemical quenching (NPQ_I_) of *Thymus carnosus* Boiss combinations, T, T-T, and T-r (**a**), and *Retama* (L) Boiss combinations, R and R-t (**b**), during the drought treatment (day 0 = before water restriction). Values are mean + SE. Significant differences between watering treatments for each combination over the entire restriction period are indicated next to the legend with different letters (uppercase for T and R, lowercase for T-T, Greek letters for T-r and R-t; two-way ANOVA; *p* < 0.05). Overall significance analysed by three-way ANOVA is indicated in lowercase black letters; *p* < 0.05.

Non-photochemical quenching (NPQ) differed between control and water restriction treatments in the T-r and R combinations, with control plants showing higher values than water restriction plants (Figure A2). When combinations were compared under water restriction, no significant differences in NPQ were detected.

### 2.3. Biomass Allocation

**Figure 6:**
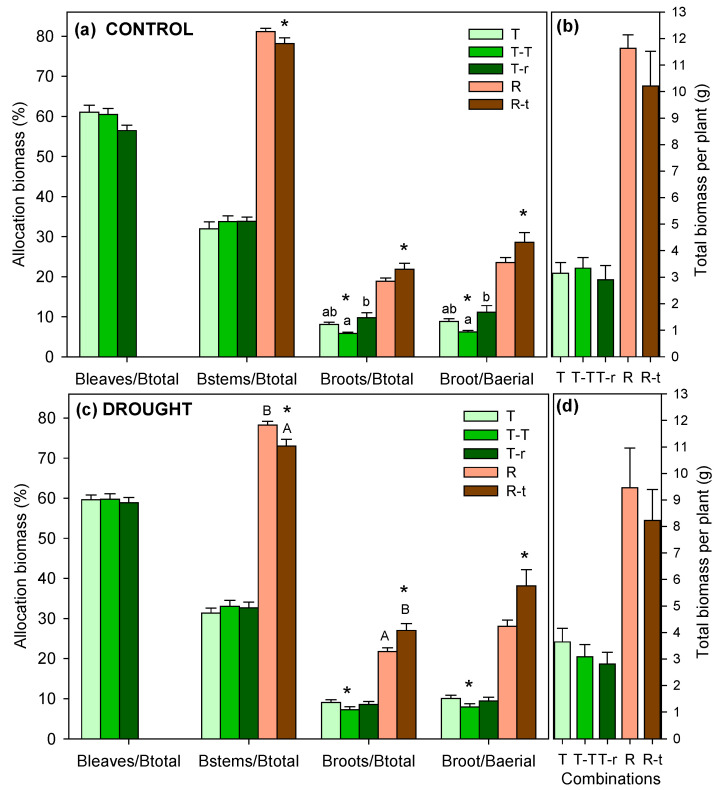
Biomass allocation in *Thymus carnosus* Boiss, and *Retama monosperma* (L.) Boiss combinations under control (**a**,**b**) and drought (**c**,**d**) treatments (mean + SE). Panels (**a**,**c**) show leaf (Bleaves/Btotal), stem (Bstems/Btotal), and root (Broots/Btotal) allocation as well as root-to-shoot ratio (Broots/Baerial), while panels (**b**,**d**) show total biomass per plant. Different letters indicate significant differences between competition treatments (lowercase letters for *Thymus* and uppercase letters for *Retama*; *p* < 0.05). Asterisks over pairs of bars in panels (**a**,**c**) indicate significant pairwise differences between control and water restriction treatments within each combination (*p* < 0.05).

The final biomass allocation in *Thymus* combinations showed little difference between drought and control treatments, except for T-T, where drought plants exhibited higher B_R_/B_A_ ratios (Figure 6). When the three combinations were compared, under control conditions, the control plants of T-r plants had higher B_R_/B_T_ and B_R_/B_A_ (9.8% and 29.7%) values than T-T plants (5.8% and 17.6%), with T plants showing intermediate values. No significant differences were observed among drought treatment plants. Two-way ANOVA confirmed significant effects of competition on B_R_/B_T_ and B_R_/B_A_ for *Thymus* (Table 3), with T–T differing from both T–r and T, which had overall higher values (post hoc result in Figure 6a).

For *Retama*, R-t plants showed differences between watering treatments, with drought plants having lower B_S_/B_T_ (73%) and correspondingly higher allocation to root biomass (B_R_/B_T_: 38.1%) compared to controls (Figure 6c). Overall values indicated lower B_S_/B_T_ for R-t, with higher B_R_/B_T_ compared to R plants (78 and 28%, respectively).

*Retama* achieved significantly higher final biomasses than *Thymus*, averaging over 2 g of roots per plant compared to 0.25 g for *Thymus* (F = 310.9, *p* < 0.001) and over 7 g of aboveground biomass (stems) compared to 2.7 g for *Thymus* (1 g in stems and 1.7 g in leaves) (F = 99.2, *p* < 0.001). No differences were found among *Thymus* combinations for any of the final biomasses (roots, stems, or leaves).

### 2.4. Leaf Chemistry Analyses

Leaf C and N content, C/N ratio, δ^13^C, and δ^15^N did not show differences between control and drought plants in any combination. However, *Thymus* plants exhibited a significantly higher leaf C content (48–50%) than *Retama* plants (43%), regardless of the competition treatment (Table 4 and Table 5). Leaf N content (1.5%) and C/N ratio (30–40) did not differ between combinations.

Plant interactions significantly affected both δ^13^C and δ^15^N values. T plants exhibited the highest δ^13^C values (−28‰) and δ^15^N values (4.1‰), while R-t plants showed the lowest values (−31.3‰ and 2.7‰). These results indicated a lower intrinsic water-use efficiency (WUEi) in R–t plants. *Thymus* plants growing with *Retama* (T–r) displayed intermediate values for WUEi and δ^15^N (Table 4 and Table 5).

## 3. Discussion

Our findings support the initial hypothesis that water restriction has a stronger physiological impact on *T. carnosus* when this species interacts with *R. monosperma*. The results suggest that underground competition for water may be a key factor driving the decline in water potential of this endangered species. While this interspecific competition does not show a significant effect on *Thymus* biomass, results suggest that intraspecific interactions seem to enhance both growth and drought tolerance in *T. carnosus*.

### 3.1. Morphological Responses

Despite having access to the same nutrient concentrations under optimal greenhouse conditions with constant watering, *Thymus* had lower growth rates than *Retama*. According to [54], plants naturally inhabiting poor soils, such as dune sands, inherently exhibit lower growth rates than those inhabiting more fertile soils, due to adaptations for conserving the scarce available resources. However, *R. monosperma*, being a N-fixing species, would be adapted to fertile soils that it fertilises, creating a positive feedback loop that promotes higher growth rates [55,56]. Moreover, in dune ecosystems with low vegetation cover, high elongation rates can provide a competitive advantage for seedlings by occupying space and excluding competing plants by shading them [27], as would be the case of *R. monosperma* over *T. carnosus. Retama* might be reflecting a different successional strategy [57], likely due to its greater nutrient acquisition capacity [54] and growth rate.

Regarding *T. carnosus* interactions, plants under intraspecific competition exhibited greater total elongation and number of branches compared to those in interspecific competition or growing in isolated plants, indicating a facilitative effect within the species. Although theory supports that intraspecific competition should be stronger than interspecific competition, our results suggest that facilitation can override competitive impacts under certain conditions. This pattern aligns with previous findings, where intraspecific facilitation has been shown to mitigate competition [58,59,60]. Using pots and fertilised soil may have influenced plant responses compared to field conditions with lower water and nutrient availability, potentially reducing abiotic stress. However, these conditions were consistent across both intra- and interspecific interactions.

### 3.2. Physiological Responses to Drought and Plant–Plant Interactions

In addition to morphological traits, we also investigated the physiological responses of plants under water-limited conditions. Our results indicate that *T. carnosus* exhibits greater resilience in water-limited environments, likely due to its lower water demand and potentially more efficient water-use strategies. Comparing the Ψ_m_ values recorded in this experiment with those reported under field conditions [51], we found that *Thymus* plants in control treatments (T, T-T, or T-r) exhibited Ψ_m_ values similar to those observed in autumn and winter under natural conditions, indicating that our experimental setup replicated field water availability during these seasons. Under water restriction, Ψ_m_ varied with plant interactions. Isolated *Thymus* maintained Ψ_m_ values comparable to autumn and winter field conditions, showing no water stress. In contrast, *Thymus* under intraspecific competition resulted in Ψ_m_ values (−1.3 MPa) similar to field isolated *T. carnosus* in summer (−1.2 MPa). However, *Thymus* under interspecific competition (T-r) experienced a stronger decline in Ψ_m_ (−2.4 MPa), exceeding the typical summer values for this association in the field (−1.5 MPa). These results suggest that *R. monosperma* intensified water limitation for *T. carnosus* under experimental conditions, consistent with field observations, where *Retama*’s water use strongly regulates local soil water balance, potentially exerting competitive pressure on coexisting species [51]. These results align with findings in [61], which reported that during periods of limited soil water, the greater competitive ability of *Stipa tenacissima* reduced the water status of *Cistus clusii*.

Despite being plants of the same age growing under similar conditions, *R. monosperma* showed a higher water demand than *T. carnosus* in all combinations, as reflected in its significantly lower Ψ_m_ values. *Retama* plants in control treatments showed less water stress (−1 MPa) than field values (−1.2 MPa) in autumn [51]. However, under water restriction and interspecific competition, they exhibited higher stress than field plants in summer (−2 MPa), regardless of *T. carnosus* presence. This suggests that *T. carnosus* had little effect on *R. monosperma* in terms of underground competition and that *Retama* struggles to cope with drought when access to deep water sources is restricted. In field conditions, *R. monosperma* can access the water table year-round [51,56], explaining its ability to withstand seasonal droughts. While pot experiments do not fully replicate natural rooting conditions, they allow the isolation of soil water deficit effects from other environmental factors such as temperature, nutrients, or light. Additionally, our water restriction treatment was applied during the growing period rather than during the seasonal summer drought, as we aimed to assess the impact of projected precipitation declines. This may explain differences with field observations, where drought typically occurs after the growth period.

As species adapted to semiarid environments, the photosynthetic apparatus of *T. carnosus* and *R. monosperma* remained unaffected under water restriction conditions, even though drought was imposed during the active growth period rather than the typical summer dry season. The observed decrease in Φ_PSII_ was fully reversible after a short period of darkness adaptation (as indicated by stable F_v_/F_m_ values), indicating that the observed photoinhibition was a photoprotective mechanism rather than a sign of photoinhibitory damage [62]. The maintenance of constant F_v_/F_m_ values above 0.75 in both species confirmed the absence of significant drought-induced damage to photosystem II [63]. This resilience aligns with previous studies on Mediterranean shrubs, such as *Phillyrea angustifolia* [64] and other labiates, like *Rosmarinus officinalis* or *Lavandula stoechas* [62]. These findings support observations from natural conditions in Mediterranean coastal dunes, where no decrease in maximum photochemical efficiency was recorded after the entire summer drought period [48].

However, interspecific competition (T-r, R-t) resulted in lower Φ_PSII_ values than those observed in isolated or intraspecific competition treatments (T-T) under control and water restriction conditions at the end of the water limitation treatment. Similar declines in photochemical efficiency under interspecific competition have been reported in Mediterranean species [65], highlighting the impact of resource partitioning and competition for belowground water. Interestingly, *T. carnosus* plants under intraspecific interaction (T-T) appeared to have a facilitative effect, as indicated by higher Φ_PSII_ values than isolated plants, a phenomenon previously observed in other Mediterranean shrubs where clustering can enhance microclimatic buffering [66,67,68]. The benefits of intraspecific interactions have also been reported to be larger in severe environments like water-limited ecosystems [69]. Overall, our results show that Φ_PSII_ was more affected by competition than by water restriction, reinforcing the role of species interactions in shaping physiological responses under water-limited environments, as seen in various arid and semiarid ecosystems [61,70].

Our experiment was conducted with plants of the same age, simulating the scenario where two seedlings of both species grow close together. The greater growth of *Retama* (both in elongation and final biomass) resulted in asymmetric resource competition, disadvantaging *Thymus* from the first year due to *Retama* exploiting larger soil volumes, with an average root biomass eight times greater. Biomass allocation patterns provided further insights into the physiological responses of *Thymus* and *Retama* under different interaction scenarios. Among all recorded biomass variables, differences were only found in those related to root allocation.

In the case of the control treatment, *Thymus* plants in the T-r combination exhibited higher root allocation, both as B_R_/B_A_ and B_R_/B_T_ ratios, than those in the T-T combination. Thus, under interspecific competition, *Thymus* allocated more biomass to roots than plants from intraspecific interaction, likely as a response to competition with *Retama*. However, the highest root investment was observed in *Retama*, regardless of interaction type. Since the root system is positively associated with the belowground competitive ability [71], the observed higher root allocation could confer *Retama* a competitive advantage during drought periods.

Although all combinations increased root allocation in response to water restriction, this shift was only significant in *Thymus* under intraspecific interaction, suggesting an adjustment in water uptake strategies. A characteristic adaptation of Mediterranean perennial scrublands to drought is the increasing allocation of biomass to roots, reducing the evaporation surface relative to the absorptive area [72]. Adjusting the root-to-total biomass ratio is an effective mechanism for controlling water loss during drought [73,74], as higher B_R_/B_T_ ratios reflect a strategy for maximising water uptake [9,75,76]. Likely due to this significant increase in root investment compared to the control treatment, the physiological status of *Thymus* under intraspecific competition was not as adversely affected by water restriction as it was under interspecific competition. This suggests that *Thymus* plants benefit from intraspecific interactions. Although intraspecific competition is generally expected to be stronger than interspecific competition [27,77,78], because individuals of the same species require similar environmental conditions, in our study, *Thymus* plants responded to drought differently depending on the type of interaction, exhibiting competitive responses under interspecific interactions but facilitative responses under intraspecific interactions. As highlighted by [39], species interactions are highly context-dependent and may shift between competition and facilitation depending on environmental conditions.

The response in root allocation was even more pronounced in *Retama*, particularly in the R-t combination (B_R_/B_T_: 27.01% in drought vs. 21.86% in control), reinforcing its greater water demand than *Thymus*. These findings highlight the importance of belowground competition in shaping *Thymus*–*Retama* interactions and suggest that soil water dynamics may be a key factor in determining competitive outcomes in Mediterranean environments. Moreover, the combined effect of *Retama’s* competition and water limitation likely altered the response of *Thymus* in the T-r combination, as root competition can limit root biomass allocation [25], potentially leading to lower Ψ_m_ values and constrained root development.

Although our experiment was conducted under Mediterranean conditions, the observed strategies in *Thymus carnosus* and *Retama sphaerocarpa* provide insight into how drought-tolerant and deep-rooted species may interact in other semi-arid ecosystems worldwide, especially as climate change alters precipitation patterns globally.

Leaf biochemistry provided additional insights into the physiological responses of *Thymus* and *Retama* under different interaction scenarios. The more negative δ^13^C of the *Retama*–*Thymus* interaction plants under water restriction (Table 4) supports this assumption. As a proxy for long-term water-use efficiency, δ^13^C integrates stomatal behaviour over time [79]. More negative δ^13^C values (depleted in ^13^C) typically indicate lower stomatal control, as observed in the R-t combination. This pattern aligns with the lower Ψ_m_ measured in these plants and suggests a limited ability of *Retama* to regulate water loss under drought conditions. These findings reinforce the idea that *Retama* does not follow a conservative water-use strategy, reflected in both its depleted δ^13^C values and lower Ψ_m_ values. Instead, it appears to tolerate drought by accessing deeper soil water in natural conditions, rather than by strict stomatal control. Although the final foliar N content was similar across all combinations, δ^15^N was lower in both T-r and R-t plants. This decrease in δ^15^N may indicate N scarcity due to greater use during growth [80] or increased nitrogen use efficiency, potentially driven by changes in soil nitrogen dynamics or shifts in nitrogen uptake strategies. For instance, ^15^N-depleted nitrogen could be transferred from mycorrhizal fungi or N_2_-fixing bacteria to the plants [81,82]. The presence of *Rhizobium* nodules on the roots of some *Retama* plants (personal observation) supports the hypothesis that N_2_ fixed by bacteria was transferred to the plants. Despite fertilisation to prevent nodulation, a N deficit could have occurred due to the higher growth rate of *Retama* under interspecific competition. This may have led to an increase in B_R_/B_T_ and B_R_/B_A_ ratios in T-r control plants to enhance nutrient uptake, as the B_R_/B_T_ ratio has been linked not only to water restriction but also to nutrient uptake efficiency [39,54,83].

## 4. Materials and Methods

### 4.1. Study Species

*Thymus carnosus* (Lamiaceae, hereafter *Thymus*) is an evergreen coastal shrub endemic to the southwestern Iberian Peninsula. It has been classified as critically endangered (CR) in Spain [84] and is in decline in Portugal [85]. It is also listed as a species of community interest under the EU Habitats Directive [86]. *Thymus* can reach 45 cm in height, with small, linear, fleshy leaves and a deep root system exceeding 1 m, allowing access to groundwater [51]. It coexists with *R. monosperma* throughout most of its range, making the study of *Retama*’s shading effects crucial for its conservation.

*Retama monosperma* (L.) Boiss (Fabaceae, hereafter *Retama*) is a leafless woody shrub that can grow up to 4 m in height and 9 m in diameter. It is a nitrogen-fixing shrub native to the southwestern Iberian Peninsula and northwestern Morocco [87]. It thrives in sandy coastal soils, particularly on stabilised and semi-stabilised dunes and marsh borders [88]. Despite being a native species, *Retama* exhibits invasive behaviour within its distribution range due to its high expansion rates and ability to modify environmental conditions [51,52,89].

### 4.2. Experimental Design

To assess the impact of drought on the interactions between *Thymus carnosus* and *Retama monosperma*, we conducted a water restriction experiment in a greenhouse at the University of Seville.

Seeds of both selected species were collected from Flecha del Rompido (Huelva), a Mediterranean climate area with winter rainfall and summer drought. The mean annual temperature was 18.2 °C, and the mean annual precipitation was 583 mm. The seeds were planted in seedling cell trays with a commercial substrate composed of peat, perlite, lime (1:1:1), and nutrients. We used a total of 1048 *Thymus* seeds and 600 *Retama* seeds. *Thymus* seeds were planted without pre-treatment, while *Retama* seeds were subjected to a 30 s boiling water bath to break dormancy and stimulate germination [90]. *Thymus* seeds exhibited a 15.5% germination rate (63 out of 1048 seeds), while even with pre-treatment, only 8.3% of *Retama* seeds germinated (50 out of 600 seeds).

The pots were maintained in the greenhouse at the Universidad de Sevilla under controlled temperature, ranging from 21 to 25 °C, and relative humidity of 40–60%. The plants received natural ambient light supplemented with halogen lamps from 8:00 to 16:00 h daily using Philips SON-T Agro 400W lamps.

To avoid *Rhizobium* nodule formation on *Retama* plants, a liquid commercial fertiliser (including N, P, K, Mg, Fe, Ca, S, Mn, and Zn) was added quarterly with watering, as nodule formation and maintenance are energetically expensive, and plants with access to combined nitrogen may not invest in nodules [91]. This ensured that pots with *Retama* did not receive an extra nitrogen supply, preventing any unbalanced results among the study pots.

### 4.3. Plant Transplanting

Five months after germination, the seedlings were transplanted into 2.2 L pots filled with the same substrate described above (peat, perlite, lime, and nutrients, 1:1:1). Mean plant size in height at transplant was 5 cm for *Thymus* plants and 12 cm for *Retama*. To determine the competition’s effect, interspecific and intraspecific interactions were established with two individuals per pot and compared with a control treatment consisting of one isolated individual per pot.

**Figure 7:**
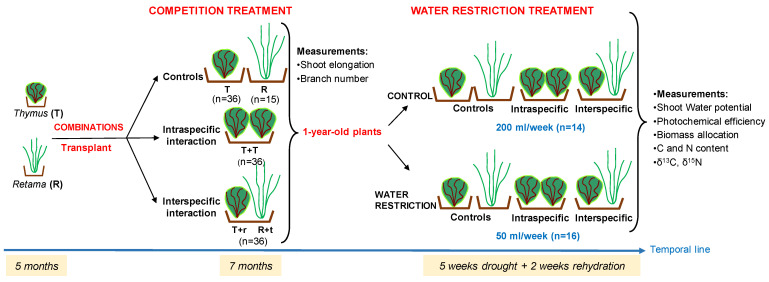
Diagram illustrating the different experimental combinations, the measurements taken, and the timeline for each phase (five months in pots after germination, seven months under different combinations, five weeks of drought, and two weeks of rehydration).

The species combinations in the pots were as follows (Figure 7):

1. Control: 36 pots with one *Thymus* plant (T) and 15 pots with one *Retama* plant (R);
2. Intraspecific competition: 36 pots with two *Thymus* plants (T-T);
3. Interspecific competition 36 pots with one *Thymus* and one *Retama* plant, (T-r or R-t). In this case, we distinguish between T–r and R–t, depending on which species was measured in the shared pot: T–r indicates measurements taken on *Thymus* growing with *Retama*, and R–t indicates measurements on *Retama* growing with *Thymus*.

In summary, a total of 123 pots were used in the experiment, with 36 pots for each combination, except for the R combination, which had 15 replicates due to the low number of *Retama* seedlings obtained. After seven months of growth under these combination conditions, the one-year-old plants were subjected to a five-week water restriction experiment. After this period, the plants were rehydrated.

Throughout the experiment, greenhouse conditions were maintained at an average temperature of 22 °C and a relative humidity of 70%, replicating the typical conditions experienced in May and November, based on the 30-year average from AEMET (1971–2000). The watering regimes were designed to simulate a moderate water deficit scenario during the active growth period, outside the typical summer drought characteristic of the Mediterranean climate. The aim was to isolate the effects of water limitation from those of extreme temperatures and to reflect future climate change projections, which predict an increase in the frequency and severity of drought events, including during typically wetter seasons. This experimental setup allows us to evaluate how reduced water availability—independent of summer heat stress—may affect plant–plant interactions and ecosystem functioning under anticipated climate change conditions.

### 4.4. Morphological Measurements

To assess changes in species growth due to interactions, the length and branching of the seedlings were measured after transplanting and before the water restriction treatment for four months. The length of each branch was measured from base to tip, and the total branch length per plant was calculated as the sum of all individual branch lengths. Additionally, the number of branches per plant was recorded.

Measurements were taken from the plants in each plot at 14–15-day intervals at the beginning of the transplant and monthly from the second month onwards. The relative elongation rate (RER) and the increase in stem branching (ΔB) were calculated as

RER = (L_t_ − L_t−1_)/Δt (1)

ΔB = B_t_ − B_t−1_ (2)

where L_t_ represents the sum of the lengths of all branches of a plant, t and t−1 refer to two consecutive measurement dates, Δt represents the number of days between these two dates, and B is the total number of branches per plant.

### 4.5. Water Restriction Experiment

The experiment was conducted in spring (April–May) to simulate drought conditions outside the typical summer dry period. The plants used in this phase were one year old, having grown for five months before transplanting and then seven months under different interaction scenarios. During this first year, all pots were watered to field capacity (FC) to ensure optimal growth.

For the water restriction experiment, two levels of watering regimes for each combination were applied: (1) drought plants (D) were watered twice a week with 25 mL each time (50 mL weekly) and (2) control plants (C, were watered twice a week with 100 mL each time (200 mL weekly) the first two weeks and up to FC for the rest of the experiment. After 35 days of water restriction treatment, when water potential values lower than those obtained under natural conditions were recorded [51], the plants were watered back to FC, and the recovery of leaf water potential values was assessed (see methods below).

Soil water content was monitored gravimetrically throughout the experiment by weighing the pots, considering the initial maximum FC recorded previously in the water treatment. This allowed us to adjust irrigation in control plants to ensure they were sufficiently irrigated. Accordingly, control plants were subjected to additional irrigation to reach FC after the second week. Since the drought experiment aimed to observe the plants’ physiological response rather than inducing mortality, we watered drought pots twice a week to keep the plants alive during the experiment, instead of eliminating watering.

Between fourteen and sixteen pots per combination and irrigation treatment were used, with 7–8 plants measured on alternate weeks to avoid consecutive measurements on the same plant. All pots were alternately distributed and rotated every week to prevent a possible greenhouse environmental effect due to differences in incident radiation or shading by neighbouring plants.

#### 4.5.1. Physiological Measurements

One week before the application of the water restriction treatment, and weekly thereafter for five weeks, midday shoot water potential (Ψ_m_, MPa), effective quantum yield (Φ_PSII_), and maximum quantum yield (F_v_/F_m_) were measured. To assess plant recovery, Ψ_m_ was measured again one week after the end of the water restriction experiment.

Chlorophyll fluorescence was measured using a portable fluorometer (mini-PAM, Walz, Effeltrich, Germany) with a pulse amplitude modulation technique. The fluorescence was excited by a pulse of modulated red light from an LED (type H-3000 Stanley) connected to a fibre optic. The maximum photochemical efficiency of PSII (F_v_/F_m_) was determined as (F_m_−F_o_)/F_m_, where F_o_ and F_m_ represent basal and maximal fluorescence of dark-adapted leaves over 20 min. Effective photochemical efficiency (Φ_PSII_) was estimated in leaves exposed to natural light conditions using Φ_PSII_ = (F’_m_−F)/F’_m_, with F’_m_ being maximal and F being steady-state fluorescence under actinic irradiance. Nonphotochemical quenching (NPQ) was calculated as NPQ = (F_m_−F′_m_)/F′_m_ [63]. Measurements were conducted from 10:00 to 12:00 h solar time on three leaves per plant, with mean values per plant used for statistical analysis.

Midday shoot water potential (Ψ_m_) was measured using a pressure chamber (Manofrigido, Lisbon, Portugal) on terminal shoots that were excised and measured immediately. Measures were taken between 12:30 and 14:00 (solar time) to record the maximum water deficit of the day when the minimum values are reached.

#### 4.5.2. Biomass Measurement

At the end of the water restriction and recovery treatment, the plants were harvested, separated into stems, leaves, and roots, and dried in a forced-air oven at 60 °C for 48 h. The final biomass of leaves, stems, and roots was determined by weighing. From the dried biomasses, the following parameters were calculated and expressed as percentages of total biomass: leaf biomass allocation (B_L_/B_T_; leaf biomass/total biomass), stem biomass allocation (B_S_/B_T_; stem biomass/total biomass), and root biomass allocation (B_R_/B_T_; root biomass/total biomass). The root-biomass-to-aboveground-biomass ratio (B_R_/B_A_) was also calculated as an indicator of the allocation pattern [92,93].

#### 4.5.3. Leaf and Cladode Isotopic Analysis and N and C Content

Five to seven leaf samples (cladodes in the case of *Retama*) from each treatment and combination (except R treatment) were collected at the end of the drought treatment to determine the carbon content (%C), nitrogen content (%N), C/N ratio, and the isotopes δ^13^C and δ^15^N. Although commercial fertiliser was used to avoid the influence of N_2_ fixation by *Retama*, its final N foliar content was analysed to confirm the absence of any significant contribution, as root nodule formation had been detected in some plants at the end of the experiment. Foliar δ^13^C was measured as a proxy for long-term intrinsic water-use efficiency (WUE_i_), based on the relationship between stomatal conductance and discrimination against the heavier carbon isotope during photosynthesis. Greater stomatal closure reduces internal CO_2_ concentration, leading to less ^13^C discrimination by Rubisco and thus higher δ^13^C and WUE_i_ values [94,95,96,97].

The cladodes and leaves collected were dried at 60 °C for 72 h and ground using a ball mill (Retsch, Haan, Germany). The samples were then combusted using an elemental analyser (Carlo Erba EA, Milan, Italy) interfaced with a continuous-flow stable isotope ratio mass spectrometer (SIRA II, VG-Isotech, Middlewich, UK). Samples were standardised to IAEA.N_2_, IAEACH-4, and IAEA-CH-6 (International Atomic Energy Agency). The isotope ratios of C and N were expressed relative to VPD (vapour pressure deficit) for δ^13^C and to atmospheric N_2_ for δ^15^N. The analytical error estimated was 0.05‰ for C and 0.2‰ for N. The isotope measurements were presented in notation δ as

δ (‰) = 1000 ∗ (R_sample_ − R_standard_)/R_standard_

where R is the isotope ratio (^13^C/^12^C and ^15^N/^14^N) of the foliage samples and the standards respectively.

The N and C content of the collected leaves was also determined from the combusted samples in the elemental analyser (Carlo Erba EA, Milan, Italy) coupled to the isotope ratio mass spectrometer.

### 4.6. Statistical Analysis

The effect of plant interaction (combinations) on relative elongation rate (RER) was assessed through repeated-measures ANOVA with time as the within-subject factor and combinations as the between-subject factor. A one-way ANOVA was performed to evaluate differences between combinations at each measurement date. Branching increment (ΔR) was analysed using the non-parametric Kruskal–Wallis and Mann–Whitney U tests. Additionally, a Pearson correlation analysis was used to explore the relationship between elongation and branch number.

Differences in pot weights during the water restriction treatment were analysed using repeated-measures ANOVA for control and drought individuals, with time as the within-subject factor and combinations as the between-subject factor, to assess differences in soil moisture between combinations over the five weeks.

The effects of plant interactions and water restriction on shoot water potential, as well as effective and maximum fluorescence, were analysed using a three-way ANOVA, with combination, watering, and time as fixed factors. Differences between drought and control plants across combinations were tested using a one-way ANOVA. Additionally, a two-way ANOVA was performed separately for drought and control treatments to assess their effect on biomass variables and C and N content, C/N ratio, δ^13^C, and δ^15^N, with combination and watering as fixed factors. Notably, repeated-measures ANOVA was not applied to physiological variables as different plants were measured on alternating weeks. Post hoc comparisons were conducted using Tukey’s test.

All data were tested for normality using the non-parametric Kolmogorov–Smirnov test. The statistical analyses were performed with SPSS v. 29.0.1 (IBM SPSS Statistic Inc., Chicago, IL, USA).

## 5. Conclusions

By experimentally restricting water, we showed that soil water availability drives both intraspecific and interspecific interactions between two shrub species. As reported in previous studies, intraspecific competition in *Thymus*–*Retama* interactions was less intense than interspecific competition. Overall, our results underscore the role of belowground competition in shaping *Thymus*–*Retama* interactions and highlight how soil water dynamics influence competitive outcomes in Mediterranean environments. Given the projected increase in drought frequency and severity due to climate change, particularly outside the summer period, our findings suggest that interactions with *Retama* seedlings may hinder the recruitment of *T. carnosus*. In contrast, well-established *Thymus* populations could facilitate seedling establishment by acting as nurse plants under favourable conditions. These results highlight how drought can alter the balance between competition and facilitation, with important implications for the conservation of endangered Mediterranean endemic shrubs. By identifying specific biotic interactions that may either constrain or support population persistence, this study contributes to a better understanding of vegetation responses to future climatic scenarios. In summary, our study contributes to a broader understanding of how species interactions and physiological traits mediate plant responses to drought. These findings may help predict vegetation dynamics not only in Mediterranean landscapes but also in other dryland ecosystems that share similar functional and climatic constraints.

## Figures and Tables

**Table 1:** Results of repeated-measures ANOVA for the factors competition and time on relative elongation rate (RER). *Thymus* Boiss and *Retama monosperma* (L.) Boiss are shown separately. Significant values (*p* < 0.05) are indicated in bold.

RER	*Thymus*			*Retama*		
	df	F	*p*	df	F	*p*
Competition	2	6.8	**0.001**	1	4.678	**0.036**
Time	4	318.5	**0.001**	4	46.332	**0.000**
Competition × Time	8	10.1	**0.001**	4	0.548	0.463

**Table 2:** Results of the three-way ANOVA for the effect of the factors competition (C), watering (W), and time (T) during the drought experiment on the variables shoot water potential (Ψ_m_), effective photochemical efficiency (Φ_PSII_), and maximum photochemical efficiency (F_v_/F_m_). *Thymus carnosus* Boiss and *Retama monosperma* (L.) Boiss are shown separately. Significant values (*p* < 0.05) are indicated in bold.

	Ψ_m_			Φ_PSII_			F_v_/F_m_		
***Thymus***	**df**	**F**	***p***	**df**	**F**	***p***	**df**	**F**	***p***
Competition	2	15.830	**0.001**	2	26.994	**0.001**	2	15.486	**0.001**
Watering	1	13.621	**0.001**	1	2.091	0.150	1	2.199	0.140
Time	5	13.089	**0.001**	4	0.954	0.434	4	20.989	**0.001**
C × W	2	8.641	**0.001**	2	3.156	**0.045**	2	0.883	0.415
C × T	10	2.805	**0.003**	8	2.630	**0.009**	8	0.454	0.887
W × T	5	5.607	**0.001**	4	5.110	**0.001**	4	5.057	**0.001**
C × W × T	10	2.437	**0.009**	8	2.424	**0.016**	8	0.229	0.985
***Retama***	**df**	**F**	***p***	**df**	**F**	***p***	**df**	**F**	***p***
Competition	1	0.240	0.625	1	0.225	0.636	1	8.335	**0.005**
Watering	1	52.657	**0.001**	1	0.000	0.983	1	0.001	0.977
Time	5	6.152	**0.001**	3	8.028	**0.001**	3	13.240	**0.001**
C × W	1	0.198	0.657	1	15.172	**0.001**	1	0.030	0.864
C × T	5	0.108	0.990	3	1.964	0.125	3	0.859	0.466
W × T	5	7.250	**0.001**	3	1.263	0.292	3	3.348	**0.022**
C × W × T	5	0.266	0.931	3	0.194	0.900	3	0.621	0.603

**Table 3:** Results of the two-way ANOVA for the effect of the factors competition (C) and watering (W) on the variables allocation of biomass to leaves (B_L_/B_T_), to stems (B_S_/B_T_), to roots (B_R_/B_T_), and root-to-aboveground ratio (B_R_/B_A_). Significant values (*p* < 0.05) are indicated in bold. *Thymus carnosus* Boiss and *Retama monosperma* (L.) Boiss are shown separately.

Thymus	B_L_/B_T_			B_S_/B_T_			B_R_/B_T_			B_R_/B_A_		
	**df**	**F**	***p***	**df**	**F**	***p***	**df**	**F**	***p***	**df**	**F**	***p***
Competition	2	1.520	0.225	2	0.881	0.418	2	7.339	**0.001**	2	6.996	**0.002**
Watering	1	0.135	0.715	1	0.467	0.496	1	0.418	0.520	1	0.287	0.593
C × W	2	0.656	0.522	2	0.021	0.979	2	1.949	0.149	2	1.988	0.143
***Retama***				**B_S_/B_T_**			**B_R_/B_T_**			**B_R_/B_A_**		
				**df**	**F**	***p***	**df**	**F**	***p***	**df**	**F**	***p***
Competition				1	5.618	**0.023**	1	5.618	**0.023**	1	4.719	**0.036**
Watering				1	5.456	**0.025**	1	5.456	**0.025**	1	4.115	**0.050**
C × W				1	0.449	0.507	1	0.449	0.507	1	0.551	0.463

**Table 4:** Results of leaf C content (%C), leaf N content (%N), C/N ratio, δ^13^C, and δ^15^N in *Thymus carnosus* Boiss and *Retama monosperma* (L.) Boiss combinations under control (C) and drought (D) treatments. Mean and standard deviation (SD) are shown. Letters denote differences between competition treatments for each variable. Lowercase letters indicate differences for control individuals, italic lowercase letters indicate differences for water restriction individuals, one-way ANOVA; *p* < 0.05).

		%C		%N		C/N		δ^13^C		δ^15^N	
Treatment		C	D	C	D	C	D	C	D	C	D
T	Mean	47.9 ab	47.9 *a*	1.5	1.4	32.8	39.7	−28.8	−27.0 *a*	5.1 a	4.1 *a*
	*SD*	*1.3*	*1.6*	*0.4*	*0.6*	*9.7*	*16.1*	*2.1*	*3.6*	*1.8*	*1.6*
T-T	Mean	49.1 a	49.0 *a*	1.7	1.5	31.8	34.5	−28.5	−28.6 *ab*	3.7 ab	3.3 *ab*
	*SD*	*1.6*	*1.1*	*0.5*	*0.4*	*9.0*	*8.2*	*2.7*	*1.1*	*0.3*	*0.6*
T-r	Mean	49.6 a	50.0 *a*	1.4	1.6	35.3	33.9	−29.1	−29.3 *ab*	2.8 b	2.6 *b*
	*SD*	*3.6*	*2.7*	*0.3*	*0.4*	*6.3*	*9.7*	*2.1*	*1.4*	*0.5*	*1.0*
R-t	Mean	42.9 b	42.9 *b*	1.4	1.3	32.9	36.2	−30.5	−31.3 *b*	2.5 b	2.7 *b*
	*SD*	*1.6*	*2.0*	*0.4*	*0.3*	*8.2*	*8.6*	*0.8*	*0.9*	*1.8*	*1.0*

**Table 5:** Results of the two-way ANOVA for the effect of combination and watering on the variables leaf C content (%C), leaf N content (%N), C/N ratio, δ^13^C, and δ^15^N on *Thymus carnosus* Boiss plants. Significant values (*p* < 0.05) are indicated in bold.

	% C			% N			C/N			δ^13^C			δ^15^N		
	df	F	*p*	df	F	*p*	df	F	*p*	df	F	*p*	df	F	*p*
Competition	3	20.886	**0.001**	3	0.956	0.423	3	0.359	0.783	3	4.212	**0.011**	3	6.698	**0.001**
Watering	1	0.038	0.847	1	0.940	0.338	1	1.554	0.220	1	0.059	0.809	1	1.114	0.297
C × W	3	0.029	0.993	3	0.632	0.599	3	0.638	0.595	3	0.684	0.567	3	0.380	0.768

## Data Availability

The original contributions presented in this study are included in the article. Further inquiries can be directed to the corresponding author.

## Abbreviations

The following abbreviations are used in this manuscript:

RER	Relative elongation rate
ΔR	Branching increment
Ψ_m_	Shoot water potential
Φ_PSII_	Effective photochemical efficiency
F_v_/F_m_	Maximum photochemical efficiency
NPQ	Non-photochemical quenching
B_L_/B_T_	Leaf mass allocation
B_S_/B_T_	Stem mass allocation
B_R_/B_T_	Root mass allocation
B_R_/B_A_	Root-biomass-to-aboveground-biomass ratio
δ^13^C	Leaf carbon isotope signature (^13^C/^12^C)
δ^15^N	Leaf nitrogen isotope signature (^15^N/^14^N)
WUEi	Intrinsic water-use efficiency
